# Assessing Cell Competition in Human Pluripotent Stem Cell (hPSC) Cultures

**DOI:** 10.1002/cpz1.435

**Published:** 2022-05-27

**Authors:** Christopher J. Price, Ivana Barbaric

**Affiliations:** ^1^ School of Bioscience The University of Sheffield Western Bank Sheffield United Kingdom; ^2^ Neuroscience Institute The University of Sheffield Western Bank Sheffield United Kingdom

**Keywords:** cell competition, culture‐acquired genetic variants, fitness‐sensing assays, fluorescent hPSC subline, human pluripotent stem cells (hPSCs), single‐cell cloning hPSCs

## Abstract

Cell‐cell interactions are required for development and homeostasis in multicellular organisms from insects to mammals. A critical process governed by these interactions is cell competition, which functions throughout development to control tissue composition by eliminating cells that possess a lower fitness status than their neighbors. Human pluripotent stem cells (hPSCs) are a key biological tool in modeling human development and offer further potential as a source of clinically relevant cell populations for regenerative medicine applications. Recently, cell competition has been demonstrated in hPSC cultures and during induced pluripotent stem cell reprogramming. In turn, these findings suggest that hPSCs can be used as a tool to study and model cell‐cell interactions during different stages of development and disease. Here, we provide a panel of protocols optimized for hPSCs to investigate the potential role that cell competition may have in determining the fate and composition of cell populations during culture. The protocols entail assessment of the competitive phenotype and the mode through which cell competition may lead to elimination of less‐fit cells from mosaic cultures with fitter counterparts. © 2022 The Authors. Current Protocols published by Wiley Periodicals LLC.

**Basic Protocol 1**: Electroporation of hPSCs to establish a fluorescent reference cell line

**Support Protocol 1**: Single‐cell dissociation of hPSCs

**Support Protocol 2**: Single‐cell cloning of fluorescently labeled hPSCs

**Basic Protocol 2**: Separate culture and co‐culture proliferation assays

**Basic Protocol 3**: Assessing levels of apoptosis in hPSC cultures using flow cytometry

**Basic Protocol 4**: Transwell assay

**Support Protocol 3**: Immunohistochemistry and image quantification of cleaved caspase‐3

**Basic Protocol 5**: Cell confrontation assay

**Basic Protocol 6**: Cell compression assay

**Basic Protocol 7**: Time‐lapse imaging to assess mechanical extrusion

## INTRODUCTION

Cell competition is a cell‐cell interaction that functions as a fitness‐sensing mechanism to detect and eliminate cells of lower fitness in comparison to their neighbors. Cells of lower fitness that are eliminated through cell competition are generally termed the “loser” population, whereas the fitter cells that survive cell competition are termed “winners” (Morata, 2021). Initially described and studied in the *Drosophila* wing imaginal disk, cell competition has since been reported in mammalian systems across a variety of contexts from development to cancer (Bowling, Lawlor, & Rodriguez, 2019). The diversity and scope of tissues in which cell competition has been described are also reflected in the mechanisms that potentially underpin the competitive phenotype (Baker, 2020). In brief, fitness‐sensing mechanisms can be broadly categorized into three main models: competition for growth factors, direct cell‐cell fitness sensing, and mechanical forces exerted through competition for space.

More recently, cell competition has been described as a mechanism of selection in both the generation of induced pluripotent stem cells (iPSCs) (Shakiba et al., 2019) and the culture of human embryonic stem cells (Price et al., 2021). Pluripotent stem cells are a unique tool that, under the appropriate conditions, can either self‐renew indefinitely or differentiate into any tissue‐specific cell type. Possession of these unique and defining features makes pluripotent stem cells an attractive tool for use in disease modeling and regenerative medicine applications.

Discovery and expansion of cell competition across new and diverse models of development and disease bring with them a requirement for a robust set of assays that can identify the presence of cell competition within a culture population and subsequently identify the determining factors that govern the competitive cell‐cell interactions observed.

**Figure 1:**
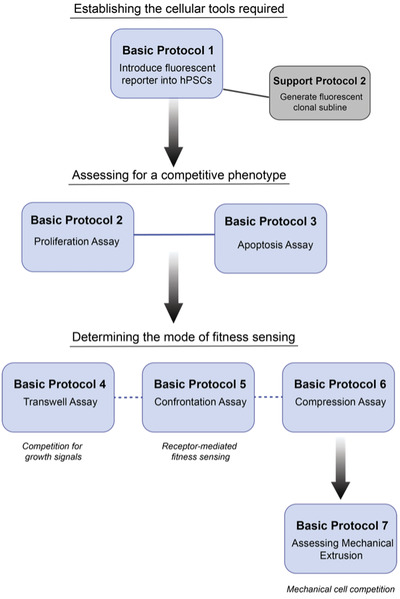
Overall protocol workflow.

*NOTE*: All solutions and equipment coming into contact with cells must be sterile, and proper sterile technique should be used accordingly.

*NOTE*: All culture incubations are performed in a 37°C, 5% CO_2_ incubator unless otherwise specified.

In this article, we provide an extensive overview of the assays required to study cell competition in human pluripotent stem cell (hPSC) cultures (see Fig. 1 for a workflow). Firstly, we provide a protocol for generating and characterizing fluorescently labeled sublines (Basic Protocol 1 and Support Protocol 2) to facilitate mixing studies. We then describe a protocol for assaying the proliferation of cell populations in separate and co‐culture conditions using a high‐content imaging approach to screen for competitive phenotypes (Basic Protocol 2). Next, we cover how to determine levels of apoptosis between potential winner and loser cells in separate cultures and co‐cultures (Basic Protocol 3). Finally, we provide protocols for testing the contribution of different fitness‐sensing mechanisms, including competition for growth factors (Basic Protocol 4), competition mediated through direct fitness sensing (Basic Protocol 5), and competition for space (Basic Protocols 6 and 7). We also provide protocols for single‐cell dissociation (Support Protocol 1) and performing immunostaining to assess apoptosis (Support Protocol 3).

## Basic Protocol 1 ELECTROPORATION OF hPSCs TO ESTABLISH A FLUORESCENT REFERENCE CELL LINE

Prior to undertaking *in vitro* investigation of competitive behavior between two cell populations, one or preferably both of the cell lines of interest should be fluorescently labeled. Fluorescent labeling facilitates distinction between the respective cell populations in the assays described later in this article. This protocol is used to generate hPSC lines constitutively expressing a fluorescent protein under control of the CAG promoter (Liew, Draper, Walsh, Moore, & Andrews, 2007).

### Materials

T12.5 flask of 40% confluent hPSCs
mTeSR1 medium (STEMCELL Technologies, cat. no. 85850)
Geltrex (Geltrex LDEV‐Free Reduced Growth Factor Basement Membrane Matrix, Thermo Fisher Scientific, cat. no. A1413202)
Dulbecco's Modified Eagle's Medium/Nutrient Mixture F‐12 Ham (DMEM/F12 medium; Merck Life Science, cat. no. D6421), 4°C
Mammalian expression vector [for expression of fluorescent marker driven by CAG promotor (Liew et al., 2007); pCAG‐H2B‐RFP (Zhang et al., 2019) and pCAG‐H2B‐GFP (Addgene, cat. no. 184777) plasmids recommended]
Y‐27632 (Generon, cat. no. A11001‐10)
Neon Transfection System (Thermo Fisher Scientific, cat. no. MPK10025), including Neon tubes and tips, buffer E2, and buffer R
Puromycin (Thermo Fisher Scientific, cat. no. A11138)

T12.5 flasks (12.5‐cm^2^ cell culture flasks)
15‐ml conical Falcon tubes
Neon Transfection System pipet station (Thermo Fisher Scientific, cat. no. MPS100)
1.5‐ml Eppendorf tubes
5‐ml serological pipets
Inverted microscope

Additional reagents and equipment for preparing single‐cell suspension (see Support Protocol 1)

*NOTE*: The confluency and medium conditions of hPSCs prior to electroporation can strongly influence the efficiency of electroporation and the survival of the cells. We find that cell cultures of 60% to 70% confluency (∼225,000 cells/cm^2^ or 2.8 × 10^6^ cells per T12.5 flask) that have been freshly fed yield good transfection efficiencies and display good survival post‐transfection.

### Day before electroporation

1 Feed a T12.5 flask of 40% confluent hPSCs with >6 ml mTeSR1 medium.

### Day of electroporation

2 Coat two T12.5 flasks with Geltrex as per manufacturer's instructions:
  - Thaw an aliquot of Geltrex on ice or overnight in the refrigerator (2° to 8°C).
  - Dilute Geltrex 1:100 in pre‐chilled DMEM/F12 medium.
  - Coat growth surface of each flask with 2.5 ml Geltrex (200 μl per cm^2^).
  - Incubate Geltrex‐coated flasks at 37°C for ≥1 hr.
3 Defrost mammalian expression vector (e.g., pCAG‐H2B‐GFP) on ice.
  Stock plasmid concentrations should ideally range between 2.0 and 5.0 μg/μl to minimize the volume added in step 8.
4 Prepare 10 ml mTeSR1 medium supplemented with 10 μM Y‐27632 in a 15‐ml conical Falcon tube and pre‐warm to 37°C.
5 Set up Neon Transfection System for electroporation:
  a. Place Neon Transfection System pipet station inside a tissue culture hood and load with a Neon tube.
  b. Fill Neon tube with 3 ml buffer E2, ensuring that the electrode is completely immersed.
6 Prepare a single‐cell suspension from a T12.5 flask of hPSCs (see step 1) that are approximately 60% to 70% confluent, as described in Support Protocol 1. At step 4 of Support Protocol 1, use 4 ml of the mTeSR1 supplemented with 10 μM Y‐27632 prepared in step 4 of this protocol.
7 Resuspend cells in buffer R at 2.5 × 10^6^ cells/120 μl and transfer 120 μl per transfection to a 1.5‐ml Eppendorf tube.
8 Pipet 5 μg of the defrosted plasmid from step 3 into the 1.5‐ml Eppendorf tube containing 120 μl cell suspension. Using a P200 pipet, gently mix cell and plasmid DNA by pipetting up and down 3 to 4 times, ensuring not to introduce any air bubbles.
9 Load a Neon tip onto the Neon pipet and place toward bottom of the 1.5‐ml Eppendorf tube containing the cell‐DNA suspension. Slowly aspirate cell‐DNA mixture into the Neon tip, ensuring that no air bubbles are present.
  The presence of air bubbles within the Neon tip will prevent the electroporation from being run. If air bubbles are visible within the tip, gently pipet the solution back down into the 1.5‐ml Eppendorf tube and allow for the air bubbles to rise to the surface of the cell‐DNA suspension. Then, re‐aspirate the cell‐DNA suspension, taking care to ensure that the Neon tip is immersed while pipetting.
10 Insert Neon pipet, with the metal head facing the electrode, into the Neon tube until a click sound is heard.
11 Transfect cells using the following conditions: Voltage: 1600 V, Pulse width: 20 msec, Pulse #: 1.
12 Remove Neon pipet from the pipet station and transfer electroporated cells to the remaining 6 ml mTeSR1 medium supplemented with 10 μM Y‐27632.
13 Aspirate excess Geltrex from the two pre‐prepared T12.5 flasks (see step 2). Using a 5‐ml serological pipet, seed 3 ml of the 6‐ml cell suspension in each flask and label flasks appropriately.
14 Place flasks in a 37°C, 5% CO_2_ incubator and manually agitate back and forth and side to side to evenly distribute cells across the growth surface. Incubate for 24 hr or overnight.

### Days after electroporation

15 Day 1 (24 hr):
  - Check for fluorescent marker expression in transfected cells using an inverted microscope.
  - Prepare mTeSR1 supplemented with 0.5× the optimal concentration of puromycin and replace medium in each T12.5 flask with 4 ml of this medium.
    The puromycin concentration should be optimized for each hPSC line used by performing a kill curve for the cell line of interest. We find that a final concentration of 0.375 μg/ml works well for many hPSC lines. A stepwise increase in the concentration of puromycin post‐electroporation (see steps 15 to 17) encourages the selection of stably transfected cells and minimizes their loss from the culture.
16 Day 2 (48 hr): Prepare mTeSR1 supplemented with 0.75× the optimal concentration of puromycin and replace medium in each T12.5 flask with 4 ml of this medium.
17 Day 3 (72 hr): Prepare mTeSR1 supplemented with the optimal concentration of puromycin and replace medium in each T12.5 flask with 4 ml of this medium.
18 Every 48 hr, refresh medium in each flask with mTeSR1 supplemented with the optimal concentration of puromycin to maintain selection while stably transfected fluorescent colonies are established.
  Confirm expression of fluorescent marker in all cells of the surviving colonies during the period of culture while the cells are undergoing selection.
  Once established, pick cultures containing entirely fluorescent colonies and expand for downstream applications. If a clonal fluorescent subline is required, follow Support Protocol 2 for single‐cell cloning.

## Support Protocol 1 SINGLE‐CELL DISSOCIATION OF hPSCs

Dissociation of hPSCs into a single‐cell suspension is required in several of the protocols within this article. This support protocol outlines the steps required to disaggregate cells and isolate a sample for cell number quantification.

### Additional Materials (also see Basic Protocol 1)

T12.5 flask of hPSCs
Dulbecco's phosphate‐buffered saline (PBS), without calcium and magnesium chloride
TrypLE Express Enzyme (Thermo Fisher Scientific, cat. no. 11528856)
Culture medium, 37°C

Automated cell counter or Neubauer improved hemocytometer (Hawksley)
Standard tabletop centrifuge

Additional reagents and equipment for counting cells (see Current Protocols article: Phelan & May, 2015)

1 Aspirate medium from the T12.5 flask of hPSCs.
2 Wash hPSCs once with 5 ml PBS per T12.5 flask.
3 Add 1 ml TrypLE Express Enzyme solution per T12.5 flask and incubate at 37°C for 3 to 4 min. Then, tap side of the flask gently to encourage cell dissociation. Assess progress of single‐cell dissociation by checking the hPSC culture under an inverted microscope after 3 min of incubation.
4 Dilute TrypLE with 4 ml culture medium. Using a 5‐ml serological pipet, gently pipet cell suspension 3 to 4 times to form a single‐cell suspension and then transfer single‐cell suspension to a 15‐ml conical Falcon tube.
5 Pipet 10 μl of the single‐cell suspension into the counting chamber of either an automated cell counter or a Neubauer improved hemocytometer (see Current Protocols article: Phelan & May, 2015).
6 Pellet cells by centrifugation for 4 min at 250 × *g* at room temperature. Aspirate most of the supernatant and gently flick tube to redisperse the cells in the remaining supernatant.

## Support Protocol 2 SINGLE‐CELL CLONING OF FLUORESCENTLY LABELED hPSCs

Following electroporation (Basic Protocol 1), it is recommended to derive clonal populations of the fluorescently labeled hPSC lines of interest. The populations generated in Basic Protocol 1 will consist of cells with differing fluorescent intensities, which may cause issues with signal detection in later protocols. The process of generating clonal sublines involves sorting single cells into a 96‐well plate using fluorescence‐activated cell sorting (FACS). This process is followed by a culture phase to expand the cells and then subsequent screening to confirm the genotype of the clonal population.

### Materials

Geltrex (Geltrex LDEV‐Reduced Growth Factor Basement Membrane Matrix, Thermo Fisher Scientific, cat. no. A1413202)
DMEM/F12 medium (Merck Life Science, cat. no. D6421), 4°C and 37°C
CloneR2 supplement (STEMCELL Technologies, cat. no. 100‐0691)
mTeSR Plus (STEMCELL Technologies, cat. no. 100‐0276), 37°C
Gentamicin (Merck Life Science, cat. no. G1397) or another widely used cell culture antibiotic
Rainbow 8‐peak alignment beads
T12.5 flasks of unlabeled and fluorescently labeled hPSCs (see Basic Protocol 1)
mTESR Plus (STEMCELL Technologies, cat. no. 100‐0276) supplemented with 10 μM Y‐27632 (Generon, cat. no. A11001‐10), 37°C

8‐channel multichannel pipet (capable of reverse pipetting)
Sterile reagent reservoirs (Starlab, cat. no. E2310‐1010)
96‐well cell culture plates
BD FACSJazz or another FACS machine
InCell Analyzer (GE Healthcare) or another fluorescent microscopy platform
Standard tabletop centrifuge with plate adapter
Inverted microscope
Geltrex‐coated 24‐well cell culture plates (see Basic Protocol 1, step 2)

Additional reagents and equipment for preparing single‐cell suspension (see Support Protocol 1), for expanding clonal lines and establishing frozen stocks, and for screening clonal lines for common genetic variants that arise during hPSC culture (see Current Protocols article: Laing, Halliwell, & Barbaric, 2019)

### 96‐well plate preparation

1 Prepare 12 ml Geltrex by diluting 1:100 in pre‐chilled DMEM/F12 medium, as per manufacturer's instructions (see Basic Protocol 1, step 2).
2 Using an 8‐channel multichannel pipet and a sterile reagent reservoir, coat each well of two 96‐well cell culture plates with 60 μl Geltrex and incubate for ≥1 hr at 37°C.
3 While the Geltrex‐coated plates are incubating, prepare 12 ml single‐cell cloning medium by adding 1.2 ml CloneR2 supplement to 10.8 ml mTeSR Plus. Supplement cloning medium with an appropriate concentration of gentamicin or another widely used cell culture antibiotic.
  Inclusion of antibiotics is required if cell sorting (steps 10 to 15) is performed under non‐sterile conditions.
4 Following incubation, aspirate Geltrex from the wells of the 96‐well plates.
5 Add 50 μl single‐cell cloning medium supplemented with antibiotics (see step 3) to Geltrex‐coated wells. Place 96‐well plates in a 37°C incubator until ready for cell sorting.

### FACS machine setup

6 Set up a BD FACSJazz or another FACS machine for cell sorting by aligning lasers and setting drop delay.
7 Using a clean, uncoated 96‐well cell culture plate, align stream deflection so that droplets are deposited into the middle of the wells.
8 Sort individual beads of uniform fluorescence (rainbow 8‐peak alignment beads) into each well of the 96‐well plate.
9 Image wells using an InCell Analyzer or another fluorescent microscopy platform to verify that the deposition and placement of single beads are correct.

### Single‐cell sorting

10 Prepare single‐cell suspensions from T12.5 flasks of unlabeled and fluorescently labeled hPSCs as described in Support Protocol 1.
11 Resuspend cells in DMEM/F12 at ∼1 × 10^6^ cells/ml.
12 Using the unlabeled hPSC cell line, set baseline fluorescence on the FACS machine.
13 Analyze a proportion of the fluorescently labeled cell population and gate those of higher fluorescent intensity for cell sorting.
14 Sort single cells across the wells of both pre‐prepared 96‐well plates (see step 5) using the following sort settings: Event rate: 500 eps, Sort setting: one drop single.
15 Centrifuge plates 1 min at 200 × *g* and return to 37°C incubator.

### Expansion of clonal lines

16 Incubate cells for 48 hr at 37°C, 5% CO_2_.
17 Day 2: After 48 hr, feed cells with an additional 100 μl single‐cell cloning medium supplemented with antibiotic solution per well. Culture cells for a further 4 days at 37°C.
18 Day 6: Under an inverted microscope, screen plates for viable hPSC colonies, which should now be visible. In the wells containing a colony, remove 120 μl old medium and replace with 120 μl fresh mTeSR Plus.
  Colonies not visible at Day 6 may continue to emerge over the next several days. It is recommended to continue checking the plates daily to spot new emerging colonies and to perform the medium changes described in steps 19 and 20 when appropriate.
19 Replenish medium every 2 to 3 days, as required, until the colonies are of sufficient size to passage.
20 When ready, manually passage individual colonies from within a 96‐well plate using a P200 tip and transfer to a Geltrex‐coated 24‐well cell culture plate containing 0.5 ml mTESR Plus supplemented with 10 μM Y‐27632.
  The Y‐27632 can be removed 24 hr after passaging.
21 Continue to expand clonal lines and establish frozen stocks using the culture system of preference.
22 Prior to use in the following protocols, screen clonal lines established above for common genetic variants that arise during hPSC culture (see Current Protocols article: Laing et al., 2019).

## Basic Protocol 2 SEPARATE CULTURE AND CO‐CULTURE PROLIFERATION ASSAYS

**Figure 2:**
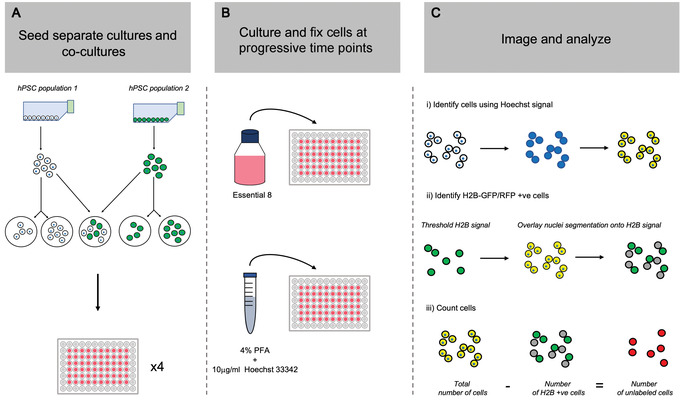
Workflow for the proliferation assays in Basic Protocol 2. (**A**) Cultures of two hPSC populations of interest are harvested and plated into separate and co‐culture conditions. (**B**) The cells are cultured for a further 96 hr, with regular medium changes and sample fixing every 24 hr. (**C**) Images are acquired and used to generate growth curves by calculating the number of cells from each population across separate and co‐culture conditions.

The first step in determining the presence or absence of cell competition between two hPSC sublines is to assess the growth rate of each cell population when cultured both in mono‐culture (separately) and within a co‐culture (mosaic) environment. The protocol requires at least one fluorescently labeled subline, generated in Basic Protocol 1, to facilitate distinguishing between the two cell populations in the co‐culture condition. However, two sublines labeled with different fluorescent markers can also be used. To test as many cell‐line pairings or culture conditions as possible, the protocol below uses a high‐content 96‐well format to maximize data acquisition. In its current format, the protocol describes the setup required to assess cell competition using a standard 3‐day culture period with equal numbers of the two cell lines. The seeding densities and length of culture period can be adapted to suit your experimental setup. Each co‐culture seeding density requires two separate culture density control conditions. In the first separate culture control, mono‐cultures equivalent to the total number of cells in the co‐culture condition (i.e., n° cells *“cell line 1”* + n° cells *“cell line 2”*) are plated. In the second separate culture control, a mono‐culture equivalent to the total number of that respective population in the co‐culture is seeded (Fig. 2).

### Materials

Vitronectin (Thermo Fisher Scientific, cat. no. A14700)
PBS, without calcium and magnesium chloride
Essential 8 medium (Thermo Fisher Scientific, cat. no. A1517001) supplemented with 20 μM Y‐27632 (Generon, cat. no. A11001‐10), 37°C
DMEM/F12 medium (Merck Life Science, cat. no. D6421), 37°C
T12.5 flasks of two different hPSC populations, with at least one population fluorescently labeled (see Basic Protocol 1)
Essential 8 medium (Thermo Fisher Scientific, cat. no. A1517001), 37°C
4% (w/v) paraformaldehyde (PFA) supplemented with 10 μg/ml Hoechst 33342 (Thermo Fisher Scientific, cat. no. H3570)

Sterile reagent reservoirs (Starlab, cat. no. E2310‐1010)
8‐channel multichannel pipet (capable of reverse pipetting)
96‐well, flat‐bottom, black, μ‐clear^®^ cell culture plates (Greiner Bio‐one, cat. no. 655090)
15‐ml conical Falcon tubes
Inverted microscope
InCell Analyzer (GE Healthcare) or another high‐content microscopy platform

Additional reagents and equipment for preparing single‐cell suspension (see Support Protocol 1)

### Plate preparation

1 Prepare a working solution of 5 µg/ml vitronectin by diluting stock aliquots 1:100 in PBS and place into a sterile reagent reservoir.
2 Using an 8‐channel multichannel pipet, add 60 μl vitronectin working solution per well to inner 60 wells of each 96‐well, flat‐bottom, black, μ‐clear^®^ cell culture plate. Incubate at room temperature for ≥1 hr.
  The electronic multichannel pipet can be used for all universal liquid handling steps.
3 After incubation, aspirate vitronectin from the wells.
  This step can be sped up by using sterile 10‐μl tips connected to an 8‐channel aspirator manifold (Merck Life Science, cat. no. BR704526).
4 Add 50 μl Essential 8 medium supplemented with 20 μM Y‐27632 to wells coated with vitronectin.
  It is important that the wells coated with vitronectin do not dry out, as this can negatively affect cell seeding. Perform step 4 quickly and, if required, divide the number of plates being handled at one time, repeating steps 3 and 4 until all 96‐well plates are completed.
5 Add 150 μl DMEM/F12 medium to the outer 36 wells of each 96‐well plate (which have not been coated with vitronectin). Place plates in a 37°C incubator until ready for cell seeding.

### Cell harvesting and plating

6 Create single‐cell suspensions of both hPSC cultures from T12.5 flasks of two different hPSC populations, with at least one population fluorescently labeled, as outlined in Support Protocol 1.
7 Resuspend cell pellets at 1 × 10^6^ cells/ml in the appropriate volume of Essential 8 medium without Y‐27632.
8 Using the suspensions from step 8, prepare suspensions at a final cell density of 3 × 10^5^ cells/ml.
  We typically add 3 ml suspension at 1 × 10^6^ cells/ml to Falcon tubes containing 7 ml Essential 8 medium and gently pipet up and down twice to ensure an even dispersion.
9 Using the suspensions from step 9, prepare suspensions at a final cell density of 1.5 × 10^5^ cells/ml.
  We typically transfer 2.5 ml suspension at 3 × 10^5^ cells/ml to Falcon tubes containing 2.5 ml Essential 8 medium and gently pipet up and down to evenly disperse.
10 Create a mosaic cell suspension at a ratio of 50:50 by mixing 2.5 ml suspension at 3 × 10^5^ cells/ml (see step 9) from each cell line in a pre‐labeled “co‐culture” 15‐ml conical Falcon tube.
11 Retrieve pre‐prepared 96‐well plates from the incubator (see step 5) and plate cells at densities of 4.4 × 10^4^ cells/cm^2^ and 2.2 × 10^4^ cells/cm^2^.
  That is, plate 50 μl of the separate (steps 9 and 10) and co‐culture (step 11) cell suspensions prepared above into their respective wells.
12 Check cells after plating under an inverted microscope and incubate for 24 hr.

### Day 0 (24 hr after plating)

13 Gently remove medium containing Y‐27632 and wash with 100 μl DMEM/F12 per well.
14 Replace medium within each well with 100 μl Essential 8 medium.
  Medium should be removed and added by slowing pipetting up or down the side of the wells to prevent the cells from lifting away from the surface of the culture plate.
15 Fix one of the plates:
  - Remove all medium from wells containing cells and wash with 100 μl DMEM/F12 per well.
  - Remove 70 μl DMEM/F12 from each well, leaving a thin layer of medium covering cells.
  - Add 100 μl of 4% PFA supplemented with 10 μg/ml Hoechst 33342 per well and incubate for 15 min at room temperature in the dark.
  - Remove 100 μl fixative solution from each well and wash with 100 μl PBS for 5 min. Repeat three additional times.
  - Aspirate DMEM/F12 from outer wells and replace it with 150 μl PBS for storage.
    Plates can be sealed with parafilm and kept in the refrigerator (4°C), protected from light, for up to 2 weeks prior to imaging.
    This post‐plating time point is considered Day 0.

### Days 1 to 3 (48 to 96 hr after plating)

16 Every 24 hr, repeat step 15, replacing culture medium with 100 μl fresh Essential 8 medium.
17 Fix a plate of cells every 24 hr, as outlined in step 16, to establish regular time points post‐plating.

### Imaging

18 After the experiment is complete, image plates using an InCell Analyzer or another high‐content microscopy platform. Capture entire well or a minimum of 16 random fields within each well. Quantify resulting images using open‐source software such as CellProfiler (Stirling et al., 2021) or Fiji (Schindelin et al., 2012) to calculate the total and individual subline cell numbers.

## Basic Protocol 3 ASSESSING LEVELS OF APOPTOSIS IN hPSC CULTURES USING FLOW CYTOMETRY

Following the use of proliferation assays to assess cell growth in separate cultures and co‐culture (Basic Protocol 2), the next step in determining the presence of a competitive phenotype is to evaluate whether the levels of apoptosis are altered between separate and co‐culture conditions. Increased levels of apoptosis in the loser population caused by the presence of the winner population are a signature of cell competition in many systems (Bowling et al., 2019). A number of approaches may be taken to assess apoptosis, including the use of live‐cell staining and in situ staining kits; however, we have found that the most consistent and versatile approach with hPSCs is to use flow cytometry. The protocol below, similar to the proliferation assays, involves establishing a co‐culture condition as well as two separate culture conditions to control for density. Cells, including those that are apoptotic and detached from the culture surface, are harvested at time points of interest and subsequently fixed and stained for the apoptosis marker cleaved caspase‐3.

### Materials

Vitronectin (Thermo Fisher Scientific, cat. no. A14700) working solution (see Basic Protocol 2, step 1)
Essential 8 medium (Thermo Fisher Scientific, cat. no. A1517001) supplemented with 20 μM Y‐27632 (Generon, cat. no. A11001‐10), 37°C
T12.5 flasks of two different hPSC populations, with at least one population fluorescently labeled (see Basic Protocol 1)
DMEM/F12 medium (Merck Life Science, cat. no. D6421), 37°C
Essential 8 medium (Thermo Fisher Scientific, cat. no. A1517001), 37°C
4% (w/v) PFA
PBS, without calcium and magnesium chloride
PBS supplemented with 0.5% (w/v) Triton X‐100
PBS supplemented with 1% (w/v) bovine serum albumin (BSA) and 0.3% (w/v) Triton X‐100
Anti‐cleaved caspase‐3 primary antibody (e.g., Cell Signaling Technology, cat. no. 9661)
Secondary antibody (e.g., Goat Anti‐Rabbit AffiniPure IgG+IgM H+L Alexa Fluor^®^ 647, Stratech, cat. no. 111‐605‐003‐JIR)

T12.5 flasks (12.5‐cm^2^ cell culture flasks)
15‐ml conical Falcon tubes
Inverted microscope
Standard tabletop centrifuge
Tube shaker (optional)
FACS tubes
BD FACSJazz or similar flow cytometry analyzer

Additional reagents and equipment for preparing single‐cell suspension (see Support Protocol 1) and counting cells (see Current Protocols article: Phelan & May, 2015)

### Plating of separate and co‐culture conditions

1 Prepare five T12.5 tissue culture flasks by coating them with 2 ml vitronectin working solution. After incubation at room temperature for 1 hr, remove vitronectin and replace with 3.5 ml Essential 8 medium supplemented with 10 μM Y‐27632. Place flasks in a 37°C incubator until ready for cell seeding.
2 Create single‐cell suspensions of both hPSC cultures from T12.5 flasks of two different hPSC populations, with at least one population fluorescently labeled, as outlined in Support Protocol 1.
3 Resuspend each hPSC subline at 1 ×10^6^ cells/ml in Essential 8 medium supplemented with 10 μM Y‐27632.
4 Prepare a dilution of each subline at 5 × 10^5^ cells/ml by adding 750 μl suspension at 1 × 10^6^ cells/ml from step 3 to a fresh 15‐ml conical Falcon tube containing 750 μl Essential 8 medium supplemented with 10 μM Y‐27632.
5 Create a mosaic cell suspension at a ratio of 50:50 by mixing 750 μl suspension at 1 × 10^6^ cells/ml from each cell line (see step 3) in a pre‐labeled “co‐culture” 15‐ml conical Falcon tube.
6 Retrieve pre‐prepared T12.5 flasks from the incubator (see step 1) and plate cells at densities of 4.4 × 10^4^ cells/cm^2^ and 2.2 × 10^4^ cells/cm^2^.
  That is, plate 550 μl of the separate (steps 3 and 4) and co‐culture (step 5) cell suspensions into their respective flasks.
7 Check cells under an inverted microscope and then place flasks in a 37°C incubator and manually agitate back and forth and side to side to evenly distribute cells across the growth surface. Incubate for 24 hr.
8 Twenty‐four hours following plating, aspirate old medium containing Y‐27632 and wash once with 4 ml DMEM/F12 medium. Replenish flasks with 4 ml Essential 8 medium without Y‐27632 and return to incubator.
9 Replace culture medium daily with 4 ml Essential 8 medium per flask.

### Processing of hPSCs for cleaved caspase‐3 staining

10 At the time point of interest, collect culture medium containing apoptotic cells that have detached from the culture flask into a 15‐ml conical Falcon tube.
11 Dissociate attached cells into a single‐cell suspension as described in Support Protocol 1.
12 Combine single‐cell suspension of previously attached cells from step 11 with the culture medium containing detached cells from step 10 and take a 10‐μl sample to perform a cell count.
13 Pellet combined sample by centrifugation for 5 min at 270 × *g*.
14 Aspirate most of the supernatant and gently flick pellet to redisperse in the remaining supernatant. Resuspend in 4% PFA at density between 1 and 5 × 10^6^ cells/ml and incubate at room temperature for 15 min.
  Use a cell density appropriate for the time point you are analyzing and the number of cells within your sample. We find that a cell suspension volume of ∼1 ml facilitates easy handling of buffer and antibody volumes in the subsequent steps. Proceed with the same cell density for the steps below.
15 Add PBS to tube to reach a final volume of 10 ml. Centrifuge fixed cells for 5 min at 270 × *g* to re‐pellet.
  Once fixed, samples can be stored in PBS prior to permeabilization for up to 2 weeks.
16 Aspirate supernatant and resuspend in permeabilization solution of PBS supplemented with 0.5% Triton X‐100 for 5 min. Centrifuge 5 min at 270 × *g* and carefully aspirate supernatant from the cell pellet.
17 Resuspend cells in blocking solution comprising PBS supplemented with 1% BSA and 0.3% Triton X‐100. Transfer one‐quarter of cell suspension per sample to a fresh 15‐ml conical Falcon tube.
  This material is to be used for the secondary‐only staining control and can be kept to the side until step 20.
18 Incubate remaining cells with anti‐cleaved caspase‐3 primary antibody diluted in blocking solution for 1 hr at room temperature or overnight at 4°C with gentle agitation.
  The antibody dilution for each batch of antibody should be determined by titration. In our experiments, anti‐cleaved caspase‐3 antibody was used at a dilution of 1:200 for flow cytometry.
19 Following incubation with the primary antibody, wash cells with 4 ml blocking solution and incubate for 5 min at room temperature prior to centrifugation for 5 min at 270 × *g*. Repeat this step three additional times.
20 Stain cells with secondary antibody in blocking solution for 1 hr at room temperature protected from light.
21 Following incubation with the secondary antibody, wash cells with 4 ml blocking solution and incubate for 5 min prior to centrifugation for 5 min at 270 × *g*. Repeat this step two additional times.
22 Resuspend cells in an appropriate volume of blocking solution and transfer to FACS tubes for analysis on a BD FACSJazz or similar flow cytometry analyzer.

## Basic Protocol 4 TRANSWELL ASSAY

**Figure 3:**
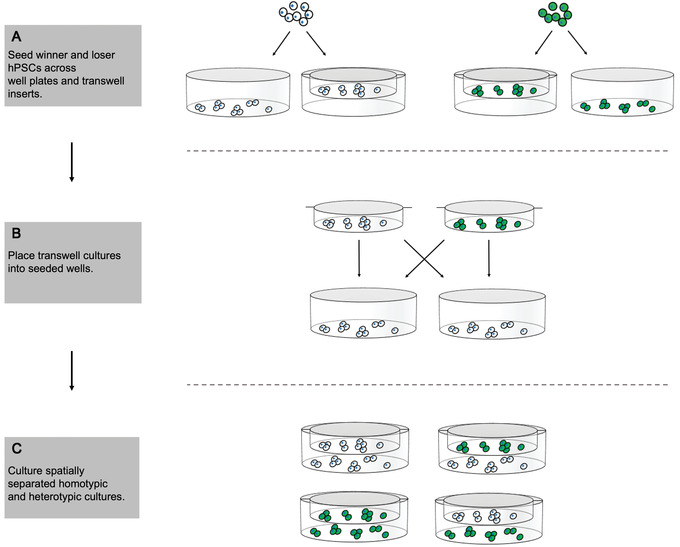
Setting up the transwell assay in Basic Protocol 4. (**A**) Prepare plates and culture inserts by seeding winner and loser hPSCs separately for 24 hr to facilitate attachment. (**B**) For each hPSC population, place half of the inserts into wells previously seeded with the same cell line (homotypic) and the other half into wells seeded with the opposing cell line (heterotypic). (**C**) Culture the spatially separated homotypic and heterotypic cultures to assess the impact of secreted factors on the competitive phenotype.

An increased death rate within the loser population that occurs due to co‐culture with winner hPSCs could be mediated through cell‐cell contacts and/or cells’ access to growth and survival factors. In this protocol, we describe how to set up a transwell assay with hPSCs that allows assessment of the contribution of secreted factors to a competitive phenotype. In a transwell setup, one cell population is grown on the bottom surface of a cell culture plate and the other on a permeable support that is suspended above the bottom of each well. The two cell populations are cultured in the same medium environment, allowing for exchange of secreted factors, but they remain physically separated for the duration of the experiment (Fig. 3). Staining for cleaved caspase‐3 is performed on winner and loser hPSCs grown with either the same or the opposing cell type in the transwell above to determine the levels of cell death in each condition.

### Materials

Vitronectin (Thermo Fisher Scientific, cat. no. A14700) working solution (see Basic Protocol 2, step 1)
Essential 8 medium (Thermo Fisher Scientific, cat. no. A1517001) supplemented with 20 μM Y‐27632 (Generon, cat. no. A11001‐10), 37°C
T12.5 flasks of two different hPSC populations, with at least one population fluorescently labeled (see Basic Protocol 1)
Essential 8 medium (Thermo Fisher Scientific, cat. no. A1517001), 37°C
DMEM/F12 medium (Merck Life Science, cat. no. D6421), 37°C
4% (w/v) PFA

Transwell inserts (Millicell Hanging Cell Culture Inserts, PET 8 μm for 24‐well plate, Millipore, cat. no. PTEP24H48)
24‐well cell culture plates

Additional reagents and equipment for preparing single‐cell suspension (see Support Protocol 1) and immunohistochemistry and image quantification of events positive for cleaved caspase‐3 (see Support Protocol 3)

1 Place 12 transwell inserts into wells of a 24‐well cell culture plate. Fill each well and transwell insert with 3 ml vitronectin working solution to prepare both culture surfaces. After 1 hr of incubation, remove inserts and place in a new 24‐well cell culture plate.
2 Aspirate vitronectin from the wells of the first 24‐well plate and replace with 0.5 ml Essential 8 medium supplemented with 10 μM Y‐27632.
3 Remove vitronectin from the transwell inserts and replace with 0.5 ml Essential 8 medium supplemented with 10 μM Y‐27632. Add a further 2 ml Essential 8 medium supplemented with 10 μM Y‐27632 to well below the insert.
  The volume of medium across the well and transwell insert will equilibrate over time, so the level of medium in the transwell may lower from that initially observed. Ensure that the level of medium does not drop to a level that exposes the vitronectin‐coated transwell membrane, as this will negatively impact cell attachment.
4 Place 24‐well plates in a 37°C incubator until ready for cell seeding.
5 Create single‐cell suspensions of both hPSC cultures from T12.5 flasks of two different hPSC populations, with at least one population fluorescently labeled, as outlined in Support Protocol 1.
6 Resuspend each hPSC subline at 1.0 × 10^5^ cells/ml in Essential 8 medium supplemented with 10 μM Y‐27632.
7 Remove 24‐well plates from step 4 from the incubator and seed 1.5 × 10^4^ cells into both each coated well and each insert, with a total of six wells and inserts per cell line.
8 Culture cells on the plate surface and in the inserts independently for 24 hr at 37°C to facilitate attachment.
9 Aspirate medium containing Y‐27632 and place inserts into the appropriate wells containing the corresponding cell line.
  That is, for each cell line, place inserts into three wells corresponding to the same cell population and three inserts into wells corresponding to the opposing cell line.
10 Add 2.5 ml fresh Essential 8 medium without Y‐27632 to each insert‐containing well.
11 Culture cells at 37°C, replacing the medium daily for a further 3 days.
12 Remove inserts from the wells and aspirate old culture medium. Wash each well once with 1 ml DMEM/F12 medium.
13 Aspirate majority of DMEM/F12, leaving a thin layer of medium coating the cells, and fix with 2 ml of 4% PFA for 15 min at room temperature.
14 Process for immunohistochemistry and image quantification of events positive for cleaved caspase‐3 as described in Support Protocol 3.

## Support Protocol 3 IMMUNOHISTOCHEMISTRY AND IMAGE QUANTIFICATION OF CLEAVED CASPASE‐3

In Basic Protocols 4 to 6, the levels of apoptosis must be evaluated using an image analysis‐based approach, as the application of flow cytometry (Basic Protocol 3) is not feasible. In this protocol, cells are fixed and subsequently stained for the apoptosis marker cleaved caspase‐3 in situ. Following staining, the cells are imaged using a fluorescent microscopy platform and quantified using open‐source software tools to calculate the proportion of apoptotic events.

### Materials

Fixed hPSCs (see Basic Protocol 4, 5, or 6)
PBS supplemented with 0.5% (w/v) Triton X‐100
PBS supplemented with 1% (w/v) BSA and 0.3% (w/v) Triton X‐100
Anti‐cleaved caspase‐3 primary antibody (e.g., Cell Signaling Technology, cat. no. 9661)
Secondary antibody (e.g., Goat Anti‐Rabbit AffiniPure IgG+IgM H+L Alexa Fluor^®^ 647, Stratech, cat. no. 111‐605‐003‐JIR)
Hoechst 33342 (Thermo Fisher Scientific, cat. no. H3570)
PBS, without calcium and magnesium chloride

InCell Analyzer (GE Healthcare) or another fluorescent microscopy platform

1 Following fixation, aspirate PFA and permeabilize fixed hPSCs in PBS supplemented with 0.5% Triton X‐100 for 10 min at room temperature.
  It is recommended that in the following steps, the volume of solution across the wells is no less than 150 μl/cm^2^.
2 Remove permeabilization solution and block with PBS supplemented with 1% BSA and 0.3% Triton X‐100 for ≥1 hr.
3 After blocking, incubate with anti‐cleaved caspase‐3 primary antibody diluted in blocking solution for either 1 hr at room temperature or overnight at 4°C.
  The antibody dilution for each batch of antibody should be determined by titration. In our experiments, anti‐cleaved caspase‐3 antibody was used at a dilution of 1:400 for immunohistochemistry.
4 Remove primary antibody solution and perform three washes with blocking solution. Incubate for 5 min per wash.
5 Prepare a secondary antibody solution containing an appropriate secondary antibody and 10 μg/ml Hoechst 33342 diluted in blocking solution.
6 Incubate cells with secondary antibody solution for ≥1 hr at 4°C protected from light.
7 Remove secondary antibody solution and perform another three washes with blocking solution. Incubate for 5 min per wash.
8 Aspirate blocking solution and replace with PBS.
  Pause point: Cells can be stored in PBS for up to 2 weeks at 4°C prior to imaging.
9 Image cells using an InCell Analyzer or another fluorescent microscopy platform.
10 Quantify number of cells and positive cleaved caspase‐3 events using open‐source software such as CellProfiler (Stirling et al., 2021) or Fiji (Schindelin et al., 2012).

## Basic Protocol 5 CELL CONFRONTATION ASSAY

**Figure 4:**
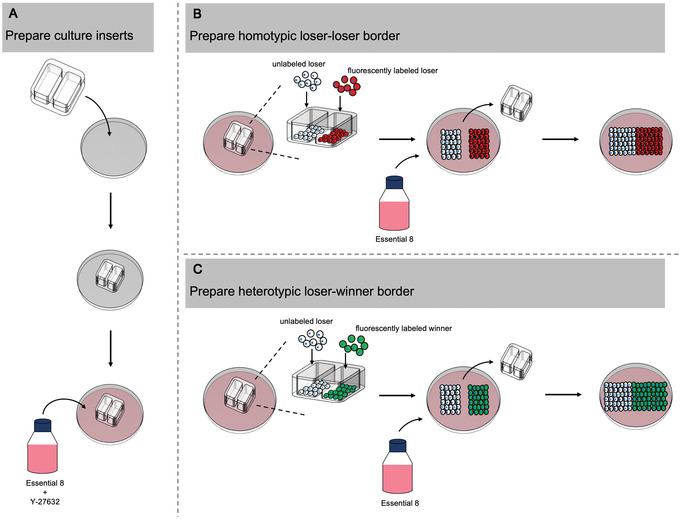
Setting up the cell confrontation assay in Basic Protocol 5. (**A**) Place a 2‐well culture insert into a well of a 12‐well plate. The inner wells of the insert are partially filled with medium supplemented with 10 μM Y‐27632, as is the remaining area of the well. (**B**) Prepare a homotypic border by seeding unlabeled and fluorescently labeled versions of loser hPSCs into separate wells of the culture insert. (**C**) Prepare a heterotypic border by seeding unlabeled loser hPSCs in one well of the culture insert and fluorescently labeled winner hPSCs in the other well. After 24 hr, remove the culture inserts from (B) and (C) and culture the cells in the wells in medium without Y‐27632 until the opposing cell fronts have been in contact for ∼48 hr.

The cell confrontation assay can be employed to distinguish between receptor‐mediated cell competition and mechanical competition. Here, two cell populations are plated onto each side of a culture insert. Removal of the insert leaves a defined gap between the populations. As the cell populations expand and migrate, they meet at a defined border (Fig. 4). The occurrence of apoptosis only at the border would be indicative of receptor‐mediated cell competition, whereas apoptosis that occurs many cell diameters away suggests mechanical cell competition. This protocol again utilizes cleaved caspase‐3 as a marker of apoptosis to identify the regions of cell death across the two cell populations after they have been cultured for a period of time following contact.

### Materials

Vitronectin (Thermo Fisher Scientific, cat. no. A14700) working solution (see Basic Protocol 2, step 1)
Essential 8 medium (Thermo Fisher Scientific, cat. no. A1517001) supplemented with 20 μM Y‐27632 (Generon, cat. no. A11001‐10), 37°C
T12.5 flasks of unlabeled loser hPSCs and fluorescently labeled loser and winner hPSCs and (see Basic Protocol 1)
DMEM/F12 medium (Merck Life Science, cat. no. D6421), 37°C
Essential 8 medium (Thermo Fisher Scientific, cat. no. A1517001), 37°C
4% (w/v) PFA

12‐well cell culture plate
Sterile tweezers
2‐well silicone inserts (Ibidi, cat. no. 80209)
Inverted microscope

Additional reagents and equipment for preparing single‐cell suspension (see Support Protocol 1) and immunohistochemistry and image quantification of events positive for cleaved caspase‐3 (see Support Protocol 3)

1 Coat center two wells of a 12‐well cell culture plate with 1 ml vitronectin working solution and incubate for 1 hr at room temperature.
2 Aspirate vitronectin and, using sterile tweezers, place a 2‐well silicone insert into center of each well. Gentle press down on corners of the inserts with either the tweezers or a sterile gloved fingertip to ensure that inserts have fully attached to the culture surface.
  If you are struggling to attach the silicone inserts to the wells, it is most likely due to incomplete aspiration of the vitronectin. We have found that a second aspiration of vitronectin from the region of the well where you are trying to place the insert will usually improve the insert attachment.
3 Add 25 μl Essential 8 medium supplemented with 10 μM Y‐27632 to each chamber of the insert.
  Ensure that the medium covers the entire surface of the well inside the chamber, as this prevents the vitronectin‐coated surface from drying out, which can negatively affect cell attachment.
4 Using a P1000 pipet, gently add 500 μl Essential 8 medium supplemented with 10 μM Y‐27632 to outer area of each well.
5 Prepare single‐cell suspensions from T12.5 flasks of unlabeled loser hPSCs and fluorescently labeled loser and winner hPSCs as described in Support Protocol 1.
6 Resuspend each hPSC subline at 1.0 × 10^6^ cells/ml in Essential 8 medium supplemented with 10 μM Y‐27632.
7 In one of the pre‐prepared wells containing inserts from step 4, plate 5 × 10^4^ unlabeled loser hPSCs in one well of the insert and 5 × 10^4^ fluorescently labeled loser hPSCs in the other.
  This condition creates a homotypic control border.
8 In the other pre‐prepared well, plate 5 × 10^4^ unlabeled loser hPSCs in one well of the insert and 5 × 10^4^ fluorescently labeled winner hPSCs in the other.
  This condition creates a heterotypic border.
  Plate 5 × 10^4^ cells, that is, 50 μl of the 1.0 × 10^6^ cells/ml suspension per well of the silicone insert.
9 Culture cells for 24 hr at 37°C to facilitate attachment.
10 Check that cells have attached properly after plating under an inverted microscope and then gently remove 2‐well silicone insert with sterile tweezers.
11 Remove medium containing Y‐27632 and wash cells once with 1 ml DMEM/F12 medium.
12 Replace with 2 ml fresh Essential 8 medium without Y‐27632 and replenish daily.
13 Culture cells for ∼4 days, monitoring the progression of the cell fronts under an inverted microscope, until the opposing cell fronts have been in contact with each other for 48 hr.
14 After the opposing cell fronts have been in contact for ∼48 hr, remove culture medium and wash once with 1 ml DMEM/F12.
15 Aspirate majority of the DMEM/F12, leaving a thin layer of medium coating the cells, and fix with 2 ml of 4% PFA for 15 min at room temperature.
16 Process for immunohistochemistry and image quantification of events positive for cleaved caspase‐3 as described in Support Protocol 3.

## Basic Protocol 6 CELL COMPRESSION ASSAY

**Figure 5:**
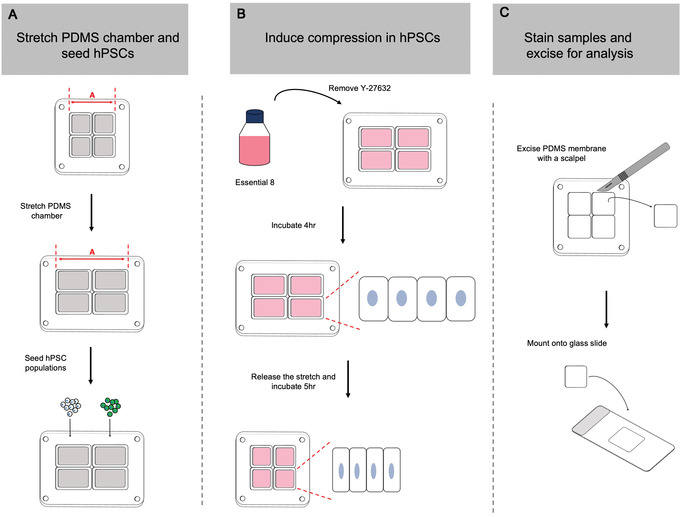
Workflow for the cell compression assay in Basic Protocol 6. (**A**) Stretch a four‐well PDMS chamber by 35% relative to its resting length. The length of the chamber is defined as distance “A.” Winner and loser hPSC populations are seeded at high and low densities into wells of the stretched chamber in medium supplemented with 10 μM Y‐27632 for 16 hr. (**B**) After 16 hr, the medium is exchanged to remove the Y‐27632, and the cells are incubated for 4 hr. Subsequently, the stretched chamber is released back to its original length to induce compression in the winner and loser hPSC populations. (**C**) After 5 hr under compression, fix the cells and process them for cleaved caspase‐3 immunohistochemistry analysis. Membranes from each well can be excised using a scalpel and mounted onto fresh glass slides for imaging.

Mechanical cell competition is underpinned by differences in sensitivity to mechanical forces between two cell populations. For example, loser cells with higher sensitivity to cell crowding and compaction are unable to tolerate the higher densities achievable by the less sensitive winner population (Price et al., 2021; Wagstaff et al., 2016). In a co‐culture scenario, this difference in compaction sensitivity leads to elimination of the loser cell population induced by cell crowding conditions. Using a compression assay originally described by Wagstaff et al. (2016), it is possible to directly test a cell line's sensitivity to compaction. In this protocol, cells are seeded onto a polydimethylsiloxane (PDMS) membrane that has been stretched in the uniaxial direction. Release of the stretched membrane back to its resting length induces compression in the seeded cells. Through analysis of apoptosis levels in compressed cells compared to their unstretched counterparts, it is possible to determine any differences between winner and loser hPSCs in their response to cell compaction (Fig. 5).

### Materials

Vitronectin (Thermo Fisher Scientific, cat. no. A14700) working solution (see Basic Protocol 2, step 1)
Essential 8 medium (Thermo Fisher Scientific, cat. no. A1517001) supplemented with 20 μM Y‐27632 (Generon, cat. no. A11001‐10), 37°C
T12.5 flasks of two different hPSC populations (see Basic Protocol 1)
DMEM/F12 medium (Merck Life Science, cat. no. D6421), 37°C
Essential 8 medium (Thermo Fisher Scientific, cat. no. A1517001), 37°C
4% (w/v) PFA
PBS, without calcium and magnesium chloride
Vectashield mounting medium (Vectashield Vibrance Antifade Mounting Medium, Vector Laboratories, cat. no. H‐1700)

Autoclave
Sterilization pouches
Four‐well PDMS stretch chambers (Strex, cat. no. STB‐CH‐4W)
Manual cell‐stretching system (Strex, cat. no. STB‐100‐10)
Petri dishes or culture plates
Calipers, electronic or dial
Inverted microscope
Ceramic tiles or thick glass slides
Thin‐bladed scalpel or razor blade
Glass slides
Tweezers
InCell Analyzer (GE Healthcare) or another fluorescent microscopy platform

Additional reagents and equipment for preparing single‐cell suspension (see Support Protocol 1) and immunohistochemistry and image quantification of events positive for cleaved caspase‐3 (see Support Protocol 3)

### Preparing the stretching chamber

1 Autoclave two four‐well PDMS stretch chambers in sterilization pouches prior to use.
2 Load one four‐well PDMS stretch chamber onto the manual cell‐stretching system and place into a petri dish or culture plate. Using calipers, measure resting length of the chamber, shown as length “A” in Figure 5A.
3 Place other four‐well PDMS stretch chamber into another petri dish or culture plate.
  This membrane will not be stretched and is treated as an uncompressed control.
4 Twist dial on the stretching system handle to stretch the PDMS chamber until it stretches by 35% relative to its resting length. Confirm correct increase in length “A” using calipers.
5 Coat each well, in both the stretched and the unstretched chambers, with 1 ml vitronectin working solution and incubate for 1 hr at room temperature.
6 Aspirate vitronectin and replace with 0.5 ml Essential 8 medium supplemented with 10 μM Y‐27632.

### Plating cells onto PDMS membranes

7 Create single‐cell suspensions of both hPSC cultures from T12.5 flasks of two different hPSC populations, as outlined in Support Protocol 1.
8 Resuspend each hPSC subline at 2 × 10^6^ cells/ml in Essential 8 medium supplemented with 10 μM Y‐27632.
9 In each four‐well PDMS chamber, seed two wells per cell line. Seed first well at a high density (between 4 × 10^5^ cells/cm^2^ and 5 × 10^5^ cells/cm^2^) to form a confluent monolayer. Seed second well at a low density (between 1 × 10^5^ cells/cm^2^ and 1.25 × 10^5^ cells/cm^2^, or 25% that plated in the high‐density condition). Adjust volume in each well to a final volume of 1 ml with Essential 8 medium supplemented with 10 μM Y‐27632.
  Seeding two wells for each population allows for both cell populations and both density conditions to be assessed in the same stretched chamber.
10 Place lid on the petri dishes or culture plates and culture cells for 16 hr at 37°C.
  The lids typically provide adequate protection from the incubator environment while allowing constant gas exchange. However, if required, antibiotics such as gentamicin or penicillin‐streptomycin can also be included in the culture medium.

### Inducing cell compression

11 Check that cells have attached to the PDMS chambers using an inverted microscope before aspirating the medium and washing each well with 1 ml DMEM/F12 medium.
12 Repeat wash as in step 11 so that the wells have been washed twice.
13 Replace medium in each well with 1 ml Essential 8 medium without Y‐27632 and incubate for a further 4 hr.
14 Release stretch on the chamber under uniaxial load by unscrewing the dial on the stretching system until length “A” of the PDMS chamber is restored to its original resting distance.
  Release of the stretch induces compaction in the seeded cells.
15 Incubate compressed and control chambers for a further 5 hr.
16 Remove majority of the medium with a P1000 pipet, taking care not to touch or damage the PDMS membrane and leaving behind a thin layer of medium coating the cells.
17 Fix with 1 ml of 4% PFA for 15 min at room temperature.
18 Process for immunohistochemistry for cleaved caspase‐3 staining as outlined in Support Protocol 3.

### Preparation of PDMS membranes for image analysis

19 After completing the secondary antibody washes in Support Protocol 3, step 7, cover a ceramic tile or thick glass slide with a thin layer of PBS before placing four‐well PDMS stretch chamber on top.
  The PBS layer prevents the PDMS membrane from sticking to the ceramic tile or glass slide during dissection of the wells in step 20.
20 Using a thin‐bladed scalpel or razor blade, cut around edge of the wells to dissect the PDMS membrane from the chamber walls.
21 Dispense 20 μl Vectashield mounting medium onto a clean glass slide.
22 Gently lift PDMS membrane away from the chamber wells using a pair of tweezers and lay onto mounting medium.
  Mount the samples with the surface containing cells face down on the mounting medium.
23 Store on a flat, dry surface for 2 hr in the dark.
24 Capture 64 random fields from each PDMS membrane using the InCell Analyzer or another fluorescent microscopy platform. Quantify resulting using open‐source software such as CellProfiler (Stirling et al., 2021) or Fiji (Schindelin et al., 2012) to identify nuclei and positive cleaved caspase‐3 signal.

## Basic Protocol 7 TIME‐LAPSE IMAGING TO ASSESS MECHANICAL EXTRUSION

**Figure 6:**
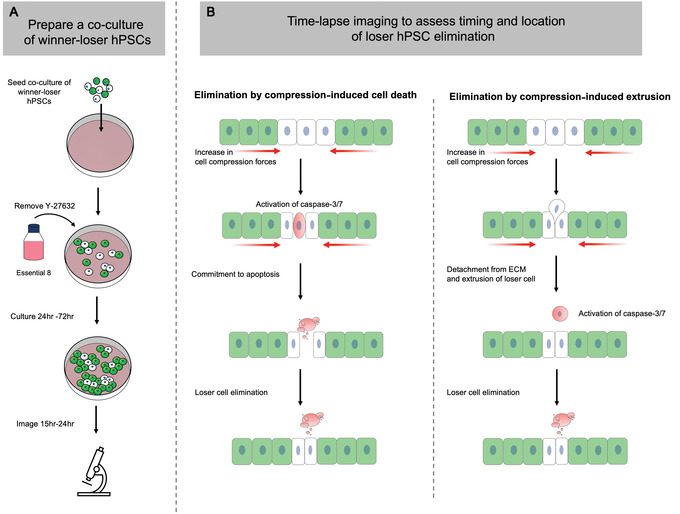
Schematic representation of Basic Protocol 7. (**A**) Establish a co‐culture of fluorescently labeled winner and loser hPSCs and culture until the time point of interest. Supplement the medium with live‐cell caspase‐3/7 dye and image on a confocal time‐lapse microscope for 15 to 24 hr. (**B**) Assess the timing of cell death and the location of cells that undergo cell death. Activation of caspase‐3/7 signal in eliminated cells prior to their removal from the culture monolayer indicates compression‐induced cell death. Alternatively, extrusion of loser cells from the culture monolayer followed by subsequent activation of caspase‐3/7 signal suggests elimination via anoikis.

During mechanical competition, the compaction forces generated can also remove loser cells via mechanisms that do not require initial onset of cell death. Instead, increased compression forces caused by cell crowding can eliminate losers by extruding them from within the monolayer, after which the cells die by anoikis (Matamoro‐Vidal & Levayer, 2019). The protocol below describes how to use confocal time‐lapse imaging and live‐cell caspase dyes to determine the timing and location of loser cell elimination during mechanical competition (Fig. 6).

### Materials

Vitronectin (Thermo Fisher Scientific, cat. no. A14700) working solution (see Basic Protocol 2, step 1)
Essential 8 medium (Thermo Fisher Scientific, cat. no. A1517001) supplemented with 20 μM Y‐27632 (Generon, cat. no. A11001‐10), 37°C
T12.5 cultures of fluorescently labeled loser and winner hPSCs (see Basic Protocol 1)
DMEM/F12 medium (Merck Life Science, cat. no. D6421), 37°C
Essential 8 medium (Thermo Fisher Scientific, cat. no. A1517001), 37°C
Reverse‐osmosis (RO) water
Essential 8 medium (Thermo Fisher Scientific, cat. no. A1517001) containing Incucyte^®^ Caspase‐3/7 Red Dye for Apoptosis (Sartorius, cat. no. 4704) diluted at 1:200

Ibidi μ‐Dish, 35 mm (Ibidi, cat. no. 81156)
15‐ml conical Falcon tubes
Inverted microscope
Zeiss LSM 880 microscope, fitted with Airyscan detection unit, cell culture chamber, and ZEN software (Carl Zeiss AG), or another confocal microscope system
500‐ml bottle
Plan‐Apochromat 40×/1.3 Oil DIC UV‐IR objective
arivis Vision4D or other appropriate software

Additional reagents and equipment for preparing single‐cell suspension (see Support Protocol 1)

### Plating a co‐culture condition for time‐lapse

1 Coat an Ibidi μ‐Dish with 1 ml vitronectin working solution and incubate for 1 hr at room temperature.
2 Aspirate vitronectin and replace with 0.5 ml Essential 8 medium supplemented with 10 μM Y‐27632. Place dish in a 37°C incubator until ready for cell seeding.
3 Prepare single‐cell suspensions from T12.5 cultures of fluorescently labeled loser and winner hPSCs as described in Support Protocol 1.
4 Resuspend each hPSC subline at 1.0 × 10^5^ cells/ml in Essential 8 medium supplemented with 10 μM Y‐27632.
5 Create a mosaic cell suspension at a ratio of 50:50 by mixing equal volumes of the suspensions at 1 × 10^6^ cells/ml suspensions from each hPSC subline in a pre‐labeled “co‐culture” 15‐ml conical Falcon tube.
  A final cell suspension volume of ≥1 ml is recommended.
6 Retrieve pre‐prepared Ibidi μ‐Dish from the incubator from step 1 and plate cells at a density of 4.4 × 10^4^ cells/cm^2^.
  That is, plate 157.5 μl of the mosaic cell suspension.
7 Check cells under an inverted microscope and then place dish in a 37°C, 5% CO_2_ incubator and manually agitate back and forth and side to side to evenly distribute cells across the growth surface. Incubate for 24 hr.
8 Twenty‐four hours following plating, aspirate old medium containing Y‐27632 and wash once with 1 ml DMEM/F12 medium. Replace with 1 ml Essential 8 medium without Y‐27632 and return to incubator.
9 Replenish culture medium daily until ready to start imaging.
  In our experimental setup using a 50:50 ratio at 4.4 × 10^4^ cells/cm^2^, cells were cultured for a further 48 hr, until Day 2.

### Day before imaging

10 Heat chamber and stage of the Zeiss LSM 880 microscope or another confocal microscope system to 37°C, place a 500‐ml bottle of clean RO water at back of the chamber, and leave to stabilize overnight.
  If it is not possible to stabilize overnight, leave for a minimum of 2 hr before mounting samples.

### Day of imaging

11 Aspirate old culture medium and wash cells once with 1 ml DMEM/F12.
12 Remove DMEM/F12 and feed cells with 1 ml fresh Essential 8 medium containing Incucyte^®^ Caspase‐3/7 Red Dye for Apoptosis diluted at 1:200.
  Protect the culture disk from light to prevent the caspase‐3/7 dye from degrading.
13 Load dish onto the microscope stage and supply with 5% CO_2_.

### Time‐lapse imaging

14 Using a Plan‐Apochromat 40×/1.3 Oil DIC UV‐IR objective, acquire a *z*‐stack of 10 μm from positions of interest starting below the central position of the nucleus and finishing beyond the apical surface. Capture fields of interest every 10 min for a 15‐ to 24‐hr period.
  The acquisition mode settings we typically use are as follows: Airyscan mode = fast, scan mode = stack, zoom = 2.0, pixel dwell = 1.81 μs, scaling X = 0.092 μm, scaling Y = 0.092 μm, scaling Z = 0.534 μm, image size: x = 106.27 μm, y = 106.27 μm. Laser powers are usually kept <2% on channels detecting H2B‐reporter fluorescence and <6% on the channel collecting live‐cell caspase‐3/7 dye signal, and the master gain = 780.
  If available, it is recommended to use a definite focus system to prevent focal drift.
15 Process raw Airyscan images in the microscope software using the auto‐strength settings.
  Processing can be done parallel to acquisition using the online mode if available.
16 Render processed data into a 4D movie using arivis Vision4D or other appropriate software.

## COMMENTARY

### Background Information

Since the first description of cell competition over 50 years ago, research into cell‐cell interactions has advanced rapidly, leading to the definition of several mechanisms that can potentially determine and define cell fate. In the classical competitive process, growth rate serves as a readout of cellular fitness (Morata & Ripoll, 1975; Simpson & Morata, 1981). Cells harboring mutations that are detrimental to cellular performance, resulting in a slower growth rate, are eliminated by the wild‐type population. Conversely, in the super‐competition paradigm, genetic changes that enhance a cell's proliferative capacity confer winner status to mutant cells, and wild‐type cells are subsequently eliminated as losers (de la Cova, Abril, Bellosta, Gallant, & Johnston, 2004; Johnston, 2014). In both contexts, the relationship between growth rate and cell competition is supported by observations that changes to the degree of difference in proliferation rates between winner and loser cells alter the intensity of the competitive phenotype (Moreno & Basler, 2004; Simpson & Morata, 1981). However, differences in growth rate are not essential for defining winner and loser status (Baker, 2020). Other factors, including metabolic activity and the differential response of pathways associated with a cell's response to stress and DNA damage, have also been extensively reported as potential measures of cellular fitness (Baker, Kiparaki, & Khan, 2019; Lima et al., 2021; Rodrigues et al., 2012). The common features and important differences between the different competitive contexts, as reviewed previously (Baker, 2020; Bowling et al., 2019), most likely reflect the evolutionarily conserved requirement for a mechanism or set of mechanisms that function across all stages of development.

Coincidently, the discovery of hPSCs before the turn of the century shaped a new field in biology to study human development. hPSCs are isolated either from cells of the inner cell mass of the pre‐implantation blastocyst (embryonic stem cells) or through reprogramming of somatic cells to the pluripotent state to generate iPSCs (Takahashi et al., 2007; Thomson et al., 1998). Following derivation, hPSCs are mostly diploid; however, during prolonged culture, hPSCs can accrue genetic abnormalities, the most common of which present as non‐random gains of chromosomal regions (Draper et al., 2004; Halliwell, Barbaric, & Andrews, 2020; The International Stem Cell Initiative, 2011). Acquisition of chromosomal abnormalities that bestow a higher proliferative ability upon the variant population subsequently provides the cells with a “winner” competitive phenotype. In mosaic hPSC cultures, cells with relatively higher proliferative abilities eliminate slower‐proliferating “loser” cells through mechanical cell competition (Price et al., 2021).

The recent establishment of a new link between cell competition and hPSCs opens new opportunities to combine the resources of two advancing fields in the study of human biology. Advances in hPSC genetic editing, robust differentiation protocols, and organoid/tissue model technology mean that it is now possible to study changes in cell phenotype at different stages of development, providing an avenue for future studies into the potential roles of cell competition in controlling the cellular composition of specific tissues.

### Critical Parameters

Cell competition can be highly context dependent, and success of the assays relies on maintaining hPSCs that are faithful to the experimental state and genotype under investigation.

#### Well‐characterized hPSC populations

Acquisition of recurrent aneuploidies that can occur upon prolonged culture of hPSCs has the potential to alter the competitive phenotype. Cultures of hPSCs should be screened regularly using karyotyping and qPCR methods to monitor for the presence of the most commonly acquired genetic changes. In addition, hPSCs can recurrently acquire other types of genetic changes, including point mutations that occur in cancer‐related loci (Avior, Lezmi, Eggan, & Benvenisty, 2021; Merkle et al., 2017). It is not yet known whether these variants lead to competitive behavior in hPSCs, but if required, more detailed genome analysis using next‐generation sequencing methods should be undertaken.

#### High‐quality hPSC cultures

Spontaneous differentiation within hPSC cultures can also impact the outcome of the assays described in this article. Exit from the pluripotent state can alter a cell's growth, physical properties, and response to external factors, all of which may influence the outcome of the assays used to characterize cell‐cell interactions. Cultures should be monitored for spontaneous differentiation by screening for pluripotency‐associated surface markers such as TRA‐160 and SSEA3 and/or transcription factors, such as NANOG, OCT4, and SOX2.

### Troubleshooting

Please see Table 1 for a troubleshooting guide.

**Table 1:** Troubleshooting Guide for Assessing Cell Competition in hPSCs

Problem	Possible cause(s)	Solution
*Basic Protocol* *1*		
Poor survival of hPSCs post‐electroporation	Prolonged incubation of cells in cytotoxic “R” buffer	Minimize time that cells spend in “R” buffer by working quickly and efficiently to perform the electroporation and dilute the buffer in the pre‐prepared mTESR medium supplemented with Y‐27632
Low transfection efficiency	Poor plasmid DNA quality; Low plasmid concentration	Purify plasmid to ensure the A_260/280_ ratio is >1.8 and the concentration is >2 μg/μl
Absence of hPSC colonies post‐selection	Puromycin concentration not optimal	Perform a puromycin kill curve to calculate the concentration required to eliminate wild‐type cells but not cause toxicity to resistant cells
*Basic Protocol* *2*		
Poor attachment of hPSCs post‐plating	Vitronectin solution is not dispersed across the well or has dried out	Ensure growth surface of the well is fully coated. Tapping the plates gently following addition of vitronectin can encourage even distribution across the well. Move quickly and efficiently when aspirating vitronectin and replacing with medium. Reduce the number of plates you are handling at one time to ensure the wells do not dry out.
Poor survival of hPSCs post‐plating	Prolonged treatment with TrypLE	Flasks with greater confluency require longer incubation with single‐cell dissociation reagents. We recommend using cultures at no greater than 60‐70% confluency (∼225,000 cells/cm^2^ or 2.8 × 10^6^ cells per T12.5 flask). Alternative single‐cell dissociation reagents such as Accutase that may be less cytotoxic upon prolonged incubation can also be used.
Cells clustering in the center of the well	Low‐speed dispensing of cells into wells; Gentle agitation of plate prior to cell attachment	Pipet cells at a moderate speed into the culture wells. We have found that this, in combination with the culture medium already present in the well, facilitates even distribution across the growth surface. Do not shake the 96‐well plates side to side and back and forth prior to incubation.
Cells lifting away during fixation	Insufficient volume of medium remaining on top of the cells	Maintain 50 μl DMEM/F12 in the wells after washing and add 50 μl 8% PFA to fix
Poor Hoechst 33342 signal	Hoechst dye has deteriorated or the concentration in PFA solution is too low	Increase the concentration of Hoechst 33342 or replace it and ensure it is stored appropriately protected from light
*Basic Protocol* *3*		
Loss of cells during staining	Speed of centrifugation is insufficient to pellet fixed cells	Increase the speed of centrifugation and/or reduce the volume of blocking solution used in the wash steps. If the volume in wash steps is reduced, it is recommended to increase the number of washes to a minimum of four.
*Basic Protocol* *5*		
Poor attachment of silicone inserts	Excess vitronectin solution remaining on the culture plate	A second aspiration of vitronectin from the region of the well where you are trying to place the insert will usually improve the insert attachment. If the problem persists, use a new clean and dry insert.
Lifting of cells following removal of the silicone insert	Cells attaching to the interior edge of the silicone insert	Lower the plating density to create a less confluent monolayer or decrease the incubation period with the insert
Poor cell expansion following removal of insert	Vitronectin has dried out	Once the insert is attached, work quickly to add medium to the outer area of each well. Prepare one well at a time if required.
*Basic Protocol* *7*		
Focal drift during imaging	Fluctuation in expansion of the microscope stage	Lengthen the stabilization time to heat the chamber and stage of the microscope. Temperatures can be checked using an infrared heat gun and adjusted where necessary. If possible, use the definite focal system at each time point to make automatic adjustments.
Unexpected cell death during imaging	Phototoxicity as a result of time‐lapse settings	Laser power may be too high for frequent time‐lapse imaging. We recommend that laser powers to detect H2B fluorescence be kept below 2% of their maximum value and below 6% on the channel collecting live‐cell caspase‐3/7 dye.
High background	Phenol red	If possible, use a medium composition without phenol red

### Understanding Results

#### Basic Protocol 1 outlines the process of electroporating cells to create fluorescent sublines

On the day following electroporation, it will be possible to view the H2B‐GFP or H2B‐RFP fluorescent signal in a proportion of the surviving cells. In the following 3 days with puromycin selection, there will be a high amount of cell death as non‐fluorescent wild‐type cells and fluorescent cells lacking sustained expression undergo apoptosis. Over the next several days, a few hPSC colonies composed of entirely fluorescent cells should emerge across the culture flask.

#### Basic Protocol 2 determines if the growth of an hPSC population is affected by the presence of another

Cell numbers for cell lines in both separate and co‐culture conditions are acquired from the quantified images on each day of the experiment. In the co‐culture condition, if using one unlabeled and one H2B‐fluorescent subline, firstly identify the total number of cells within each field using the Hoechst DNA stain. The number of cells in the fluorescently labeled population can then be identified based on their H2B‐GFP or H2B‐RFP signal. The cell number of the non‐fluorescent population is determined by subtracting the number of H2B‐positive cells from the total cell count.

Using the cell numbers, growth curves for both hPSC populations can be generated for separate and co‐culture conditions.

Findings that would indicate cell competition are as follows:

1. The number of cells from one population is lower in the co‐culture condition than in the separate culture control.
2. In the other population, the number of cells in co‐culture is either equal to or greater than the number observed in the separate culture condition.

If no difference between the number of cells found in separate culture conditions is observed in *both* hPSC populations, that indicates that cell competition is absent with those culture parameters.

#### Basic Protocol 3 determines if the level of apoptosis within an hPSC population is affected by co‐culture

In cell competition, loser cells are eliminated from the co‐culture condition by the presence of winners. Therefore, the hPSC population that displayed diminished growth rate upon co‐culture in Basic Protocol 2 should also show increased levels of cleaved caspase‐3 staining in co‐culture compared to separate culture. In contrast, cleaved caspase‐3 levels in the other hPSC population will be unaffected.

Collectively, the findings from Basic Protocols 2 and 3 will indicate if cell competition is present and which of the two hPSC populations possesses the winner or loser phenotype.

#### Basic Protocol 4 assesses the role of secreted signals in mediating a competitive phenotype

Competition for growth factors and secreted signals is one of three potential fitness‐sensing mechanisms. In the transwell assay, cleaved caspase‐3 staining levels that are increased when loser cells are cultured with winner hPSCs above them would indicate that secreted signals contribute to the competitive phenotype. If no difference in cleaved caspase‐3 levels is observed, then mechanisms that rely on cell‐cell contact are likely to define cell fitness status instead of secreted factors.

#### Basic Protocol 5 distinguishes between receptor‐mediated cell competition and mechanical competition

The cell confrontation assay should create a defined border between winner and loser hPSC populations. If, after meeting, cleaved caspase‐3 staining is higher in loser cells *only* at the border with winners, this result would suggest receptor‐mediated cell competition. Alternatively, should cleaved caspase‐3 staining be elevated within the loser population many cell diameters away from the border, this result would be indicative of mechanical competition.

#### Basic Protocol 6 assesses hPSC sensitivity to cell compaction forces

Elevation in cleaved caspase‐3 levels observed among hPSCs on compressed membranes compared to unstretched controls indicates a sensitivity to compaction forces. The greater the fold change following compression over basal apoptosis levels, the more sensitive a population is. The mechanically superior winner cells that are less sensitive to cell compaction forces should show smaller changes in apoptosis levels compared to loser cells.

#### Basic Protocol 7 identifies if the elimination of loser cells during mechanical competition is mediated by cell extrusion or cell death

Caspase‐3/7 staining observed in loser cells while they are still present within the monolayer would indicate that cell death occurs to eliminate loser hPSCs from the monolayer. In contrast, if the fluorescent signal from loser cells is observed to rise above and beyond the plane of the other cells and then co‐localize with caspase‐3/7 staining, this sequence of events would indicate crowded cell extrusion and subsequent death by anoikis.

### Time Considerations

#### Basic Protocol 1

Electroporation with the Neon transfection system takes approximately 1 to 2 hr, including time allocated for flask preparation and plasmid defrosting. Selection of cells with sustained fluorescent marker expression takes a further 5 days. Subsequent expansion of selected cells into a working subline takes between 1 and 2 weeks.

#### Support Protocol 1

Preparing a single‐cell suspension of hPSCs should take ∼12 min to complete.

#### Support Protocol 2

Preparing a clonal subline from the fluorescent population selected for in Basic Protocol 1 using single‐cell cloning will take an additional 2 to 3 weeks.

In total, the time required to generate a fluorescent subline is between 2 and 5 weeks.

#### Basic Protocol 2

Preparing and plating cells take ∼2 hr, depending on the number of cell lines or culture parameters being tested. On subsequent days, medium changes and fixation should take ≤1 hr. The total length of the experiment using the culture conditions from Price et al. (2021) is 5 days.

#### Basic Protocol 3

The culture of separate and co‐culture conditions can take up to 5 days depending on the time points of analysis chosen. Staining and analysis for cleaved caspase‐3 take a further 2 to 24 hr depending on the length of primary antibody incubation.

#### Basic Protocol 4

Co‐culture experiments in the transwell assay take ∼2 hr to plate, and medium changes take ≤1 hr on subsequent days. The length of the experiment can vary depending on the culture parameters chosen; using the conditions from Price et al. (2021) described here, the experiment takes 5 days to complete.

#### Support Protocol 3

Immunostaining should take between about 4 and 24 hr to complete.

#### Basic Protocol 5

Plating opposing cell fronts takes ∼2 hr. Following removal of the insert after 24 hr, meeting of cell fronts and growth in contact for 48 hr take ∼4 days. The total length of time for the assay to run is ∼5 days.

#### Basic Protocol 6

The cell compression assay should take ∼26 hr to complete.

#### Basic Protocol 7

Preparation of co‐culture samples for imaging should take ∼2 hr for plating, and medium changes take <1 hr on subsequent days. The length of the experiment using co‐culture parameters from Price et al. (2021) is up 5 days, with imaging performed over a 15‐ to 24‐hr window within that period.

### Author Contributions

**Christopher J. Price**: Conceptualization, Writing — original draft, Writing — review and editing; **Ivana Barbaric**: Conceptualization, Funding acquisition, Supervision, Writing — review and editing.

### Conflict of Interest

The authors declare no conflict of interest.

## Data Availability

Data sharing is not applicable to this article as no new data were created or analyzed in this study.
